# Locus coeruleus degeneration is associated with cortical tau deposition and cognitive decline in older adults at familial risk of Alzheimer's disease

**DOI:** 10.1002/alz.71427

**Published:** 2026-04-24

**Authors:** Alfie Wearn, Kate M. Onuska, Hayley R. C. Shanks, Rahul Gaurav, Stéphane Lehéricy, Giulia Baracchini, Colleen Hughes, Jennifer Tremblay‐Mercier, Sridar Narayanan, John Breitner, Judes Poirier, Taylor W. Schmitz, Gary R. Turner, R. Nathan Spreng, for the PREVENT‐AD Research Group

**Affiliations:** ^1^ Montreal Neurological Institute, Department of Neurology and Neurosurgery McGill University Montreal QC Canada; ^2^ Schulich Medicine and Dentistry, Western University London ON Canada; ^3^ Robarts Research Institute Western University London ON Canada; ^4^ Paris Brain Institute—ICM Center for NeuroImaging Research—CENIR Sorbonne Universite CNRS, Inserm Paris France; ^5^ Department of Psychological and Brain Sciences Indiana University Bloomington Bloomington Indiana USA; ^6^ Douglas Mental Health University Institute Verdun QC Canada; ^7^ McConnell Brain Imaging Centre McGill University Montreal QC Canada; ^8^ Department of Psychiatry McGill University Montreal QC Canada; ^9^ Department of Physiology and Pharmacology Western University London ON Canada; ^10^ Department of Psychology York University Toronto ON Canada; ^11^ Department of Psychology McGill University Montreal QC Canada

**Keywords:** amyloid, Alzheimer's disease, cognitive decline, healthy aging, locus coeruleus, longitudinal, MRI, neurodegeneration, neuromelanin, noradrenergic system, pathogenesis, PET, tau

## Abstract

**INTRODUCTION:**

The locus coeruleus (LC) is among the first sites of tau pathology in Alzheimer's disease (AD) and may seed neocortical tau.

**METHODS:**

We used longitudinal neuromelanin‐sensitive MRI to assess LC integrity in vivo in a cohort of cognitively unimpaired older adults with familial risk of AD in relation to tau and amyloid positron emission tomography (PET) and long‐term cognitive trajectories.

**RESULTS:**

We showed that both LC integrity at baseline and its rate of degeneration over time independently predicted a neocortical pattern of tau deposition. In keeping with the known function of the LC, neuropsychological tests showed that LC integrity at baseline predicted changes in attention. Finally, we found that longitudinal LC degeneration correlated with memory decline in people with elevated neocortical amyloid burden.

**DISCUSSION:**

Our findings underscore the importance of LC in AD pathogenesis. Longitudinal measurement of LC degeneration may help distinguish trajectories of age‐related cognitive decline and early AD.

## Highlights

- Longitudinal neuromelanin MRI was used to assess locus coeruleus (LC) integrity in vivo.
- Pre‐symptomatic Alzheimer's disease (AD) may underlie supposed “age‐related” LC degeneration.
- Faster LC degeneration predicted neocortical tau extent in people with high amyloid.
- Lower LC integrity at baseline predicted decline in attention performance.
- Faster LC degeneration predicted faster memory decline.

## 1 BACKGROUND

The locus coeruleus (LC), a tiny nucleus of noradrenergic cell bodies in the brainstem, is among the first sites of tau pathology in Alzheimer's disease (AD).^1^, ^2^ Pre‐tangles appear in the LC decades before medial temporal lobe atrophy and onset of cognitive symptoms.^3^, ^4^ Together with other neuromodulatory nuclei of the ascending arousal system (e.g., cholinergic basal forebrain), the LC is therefore a plausible origin point for tau pathology in the brain,^5^, ^6^ as detailed by the recently formalized *neuromodulatory fragility hypothesis*.^7^ According to this hypothesis, the long, highly branched axonal projections of these neurons may make them particularly vulnerable to damage,^6^, ^8^, ^9^ and may enable tau seeding to cortical and subcortical targets.^10^, ^11^ Selectively strong projections provide a plausible route for tau spread from LC to basal forebrain, entorhinal cortex, and hippocampus, before more widespread neocortical involvement in the archetypical Braak stages.^2^, ^3^, ^12^ In contrast, the LC is relatively spared from degeneration in healthy aging.^13^, ^14^, ^15^, ^16^, ^17^ It remains debated whether associations between LC degeneration and age reflect true aging effects or incipient AD pathology.^18^, ^19^, ^20^ Clarifying LC contributions to healthy versus pathological aging is therefore critical for understanding early AD pathogenesis and progression.

The LC noradrenergic system helps sustain and modulate attention,^21^, ^22^, ^23^, ^24^, ^25^ and is implicated in processes such as learning, memory, and decision‐making.^26^, ^27^, ^28^, ^29^ In older age, LC health may act as a bottleneck, whereby preserved LC integrity supports resilience across multiple cognitive domains and may represent a substrate of cognitive reserve.^30^, ^31^ When AD pathology emerges, this neuromodulatory support is progressively compromised. Compensation may initially mask deficits; clinically apparent deficits often manifest first as episodic memory impairment, reflecting medial temporal damage and disrupted noradrenergic modulation.^32^ Attentional impairment can also disrupt new information encoding, indirectly affecting memory. Dissociating natural inter‐individual variation in LC integrity from pathological degeneration is essential for distinguishing healthy aging from AD trajectories. One approach is to investigate how longitudinal trajectories of LC integrity relate to distinct cognitive domains (e.g., attention vs memory).

Neuromelanin‐sensitive MRI enables in vivo assessment of the LC integrity in older adults.^33^, ^34^, ^35^ Regions with high concentrations of neuromelanin‐containing neurons appear hyperintense, although the precise contributors to this signal are under debate.^34^, ^36^ Neuromelanin, which is thought to sequester neurotoxic by‐products of catecholamine production, is found almost exclusively in catecholaminergic cell bodies such as the LC.^37^, ^38^ Neuromelanin accrues through early and middle adulthood, peaking around age 60.^39^, ^40^, ^41^, ^42^ Thereafter, LC neuromelanin signal correlates closely with neuronal density in unimpaired older adults and people with AD,^43^, ^44^ supporting its use as a marker of structural integrity. Neuromelanin MRI‐based measures of LC integrity appear to predict neocortical tau pathology^43^, ^45^, ^46^, ^47^, ^48^ and may be useful for tracking disease onset and progression.

However, relatively few studies have tracked LC integrity longitudinally. Prior work suggests that LC pathology may precede medial temporal tau,^46^ and that LC degeneration accelerates in older age (≈70 years).^49^ These studies highlight the value of in vivo tracking of early LC changes, but do not establish whether the rate of LC degeneration provides relevant information beyond a single measure of integrity. Here, we leveraged neuromelanin MRI with amyloid beta (Aβ) and tau positron emission tomography (PET), alongside longitudinal multidomain neuropsychological testing to study how LC integrity and decline relate to AD pathology and cognition in older adults with a strong family history of AD who were cognitively unimpaired at baseline. We hypothesized that (1) LC integrity would be lower in older (vs younger) adults, but this association would be explained by the presence of incipient AD pathology; (2) lower baseline LC integrity and steeper rates of LC degeneration would be associated with greater neocortical tau deposition in regions corresponding to early Braak stages, especially in individuals with higher Aβ burden; and (3) lower baseline LC integrity and steeper rates of LC degeneration would be associated with steeper trajectories of cognitive decline, showing associations in the specific domains of memory and attention.

**RESEARCH IN CONTEXT**

1. **Systematic review**: We review the existing literature on the role of locus coeruleus (LC) in Alzheimer's disease (AD), including knowledge regarding its histologic measurement versus in vivo assessment with neuromelanin MRI.
2. **Interpretation**: We show that the rate of LC degeneration predicts the extent of neocortical tau as well as the rate of cognitive decline, independent of baseline LC integrity. In neuropsychological tests, baseline LC predicted long‐term attention decline, whereas longitudinal LC degeneration predicted memory decline. These observations suggest that longitudinal measures of LC integrity may help distinguish normal cognitive aging from changes attributable to incipient AD.
3. **Future directions**: Future work should assess the possible causative nature of LC degeneration in AD, and whether improving LC health improves disease outcomes. The role of LC degeneration in different AD subtypes should also be explored. More work is needed to understand the interactions between LC and other neuromodulatory systems and their possible role in AD pathogenesis.

## 2 METHODS

### 2.1 Participants

We used data from PREVENT‐AD, a longitudinal cohort study of older adults with a parent or multiple siblings with clinically diagnosed AD.^50^, ^51^ After exclusions detailed below, we studied 199 participants (mean age: 68.3 ± 5.2, 69.8% female) having multiple available time points of neuromelanin MRI and valid cognitive test data. Participants were followed up for an average of 46.5 ± 14.9 months. A subsample of 178 participants (mean age: 68.3 ± 5.1, 70.0% female) also had PET assessment of Aβ and tau on at least one time point, as described below.

All participants provided written informed consent, and all PREVENT‐AD study features were approved by the McGill Institutional Review Board (IRB) and/or the *Comité d’éthique de la recherche du CIUSSS de l'ouest de l'ile de Montréal*. All procedures complied with the ethical standards of the 1964 Declaration of Helsinki.

### 2.2 MRI acquisition

MRI scans were acquired on a 3T Siemens PrismaFit at the Douglas Research Centre, including a T1‐weighted anatomic scan (magnetization‐prepared rapid acquisition gradient echo [MPRAGE], 1 mm^3^ isotropic resolution, repetition time/echo time/inversion time [TR/TE/TI]  =  2300/2.96/900 ms, flip angle [FA]  =  9°, acquisition time [TA] =  5:30), and a “neuromelanin‐sensitive” turbo spin‐echo (TSE) axial slab of the brainstem and midbrain positioned perpendicular to the wall of the fourth ventricle, with the inferior boundary of the stack aligned with the fastigial recess, ensuring full coverage of the LC: 0.7 × 0.7 × 1.8 mm, TR/TE = 600/10 ms, FA = 120°, TA = 8:27, 20 slices, 7 online averages.

MRI was collected in three distinct waves between 2019–2024. No changes to the scanner hardware or software were made during this period. Participants (*n* = 269) had between one and three scans including neuromelanin MRI. Of these, 207 participants had at least two time points of neuromelanin MRI data and were therefore included in this study (mean interscan interval: 28.8 ± 6.4 months; mean total follow‐up time: 46.5 ± 14.9 months; average number of scans: 2.6 ± 0.5).

### 2.3 PET acquisition

Participants underwent Aβ ([^18^F]NAV4694), and tau ([^18^F]flortaucipir) PET. Scans were acquired on a brain‐dedicated high‐resolution research tomograph (HRRT) scanner at the McConnell Brain Imaging Centre of the Montreal Neurological Institute‐Hospital.

For Aβ PET, 6 mCi radiotracer was injected and the scan was acquired in the time window 40–70 min post‐injection. For tau PET, ≈10 mCi was injected and the scan was acquired in the time window 80–100 min post‐injection. Frames of 5 min were acquired. Images were reconstructed using an iterative reconstruction method (OP‐OSEM, 10 iterations, 16 subsets). Images were motion and decay corrected, and scatter correction was performed using a three‐dimensional (3D) method.

Two waves of PET data collection occurred, the first in 2016–2017, and the second between 2019 and 2023. For participants with multiple PET scans, images were processed by aligning the temporally closest PET‐MRI scans to minimize anatomic differences between images; however, in order to maximize our sensitivity to AD pathology, we related demographic, behavioral, and MRI variables to PET measures from participants’ latest PET session, which was often in‐line with the second wave of MRI collection. Therefore, for all analyses, baseline MRI preceded PET collection by an average of 208 ± 528 days.

### 2.4 Image processing

#### 2.4.1 Measuring LC integrity—neuromelanin MRI

**FIGURE 1:**
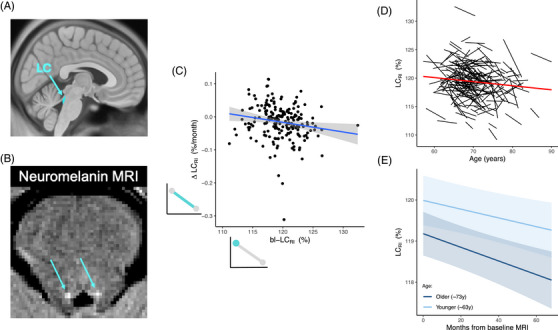
LC visualizations, longitudinal change, and association with age. (A) LC anatomic placement on sagittal Montreal Neurological Institute (MNI) space brain. LC mask shown is from Ye atlas^52^ for visualization only. (B) Neuromelanin MRI in the axial plane of a single participant with LC hyperintensities indicated by arrows. (C) Association between LC integrity at baseline and its rate of degeneration. The panel includes small schematics representing LC baseline and longitudinal change adjacent to their respective axes of the main plot. These schematics are used in figures throughout this article. (D) Longitudinal trajectories of LC integrity as a function of age in individual participants. Group trend line shown in red. (E) Longitudinal trajectories of LC integrity in younger (≈63 year) versus older (≈73 year) individuals. In the model, the interaction term for age was non‐significant, which is apparent in the figure as slopes are similar between age groups. Note that age is displayed here using binary groups for visualization purposes only; analyses incorporated age as a continuous variable in all instances (“younger” group here is represented as 1 SD below the mean; “older” as 1 SD above the mean).

At each time point, we calculated the ratio of neuromelanin‐MRI signal intensity in the LC (Figure 1A, B) to that of an adjacent pontomesencephalic reference region as a marker of structural integrity (shown to correlate with neuronal density^43^, ^44^). We calculated LC integrity using an automated pipeline described previously.^53^ First, three anatomic regions were defined *a prior*i in standard space (Montreal Neurological Institute [MNI] ICBM152 asymmetrical 2009a^54^) (Figure S1). These regions included two bilateral pontine regions, respectively, containing left and right LC (each providing a search space for individual LC signal, ≈700 mm^3^ each), and one reference region in the rostral pontomesencephalic area (≈6200 mm^3^). Using search space masks for LC, with exact localization performed using individualized “neuromelanin signal,” ensures that delineation of individual LC is robust to small alignment inaccuracies or anatomic variation between individuals. All registrations were visually inspected to ensure proper alignment with the hyperintense LC signal and to exclude visible artifacts. Next, these masks were resampled onto the neuromelanin‐sensitive scan of each individual. Using Advanced Normalization Tools,^55^ neuromelanin‐sensitive scans were rigidly aligned with concurrent MPRAGE scans, which were in turn warped to standard space using rigid and non‐linear transforms. These warps and transform matrices were then used to transform the masks from standard space to neuromelanin MRI space, using nearest‐neighbor interpolation to preserve region boundaries. Each slice of the neuromelanin‐sensitive image was then normalized such that the mean of the reference region in that slice was equal to an arbitrary value of 100. This had the dual effect of (1) correcting for slicewise variation in signal driven by scanning technique and (2) fixing all intensity values in the image relative to the reference region. We then extracted the 10 brightest (highest signal intensity) contiguous voxels on the reference‐normalized neuromelanin‐sensitive scan from within each of the left and right LC search space masks, producing a 10‐voxel mask of each LC (confirmed by visually checking all automatically produced masks). There were no other specific restraints on the LC mask shape. Finally, relative intensity of the LC (LC_RI_) was calculated by averaging signal intensity across all normalized LC voxels. Left and right LC intensities were averaged for all analyses.

Scans with mislabeled regions or artifacts on the region of interest were excluded from further analysis. A total of 24 sessions across 21 participants were excluded due to masking errors or image artifacts. Following the exclusion of these time points, eight participants no longer had two time points of valid LC measurement and were therefore excluded from the main sample (total sample size after exclusions: 199). In addition, we checked for a consistent shift in the placement of LC masks between baseline and follow‐up sessions that might explain any apparent signal changes observed. We observed almost identical overlap between group‐average masks (Figure S2), supporting any changes in LC signal as due to real change in LC integrity.

#### 2.4.2 Measuring AD pathology—PET

PET processing was performed in MATLAB (v2024b) using SPM (v25.01.02) and CAT12 (v12.9), using this pipeline: https://github.com/hayleyshanks/Longitudinal‐MRI‐PET‐preproc ^56^). We previously created an MRI population template representative of older adult brains using 3T T1–weighted data from 424 participants from the Alzheimer's Disease Neuroimaging Initiative (ADNI) cohort (as described in Ref. 56). First, each MPRAGE image in our study cohort was linearly co‐registered to the ADNI template brain using SPM's Estimate tool. These images were then segmented into gray matter (GM), white matter (WM), and cerebrospinal fluid (CSF) compartments using CAT12. Mean image quality rating was 86.6% ± 0.20%. No segmentations fell below a threshold of 70% that warranted further examination. GM and WM segmentation images (rigid‐aligned to template) were then used to calculate non‐linear warps to the ADNI population template using geodesic shooting.^57^

All frames of PET images (four for tau, six for Aβ) were aligned to the final image of the series to account for between‐volume motion and then averaged. The PET image was then co‐registered to its temporally closest MPRAGE (native‐space) using SPM's Estimate & Reslice tool. PET data were then warped to the population template brain with warps previously calculated using the MPRAGE images. Following this process, two participants were excluded for having poor alignment with the MPRAGE. We then calculated standardized uptake value ratio (SUVR) for all brain voxels relative to inferior cerebellum GM for tau and whole cerebellum GM for Aβ. Partial volume error correction was performed for both tracers and was calculated using Müller‐Gartner method with the PETPVE toolbox^58^ (default settings except GM threshold was changed from 0.5 to the slightly more liberal 0.3).

To assess global Aβ burden in each individual, we calculated a volume‐weighted mean of SUVR in the following brain cortical regions from the Desikan‐Killany atlas: Caudal Middle Frontal, Lateral Orbitofrontal, Medial Orbitofrontal, Pars Opercularis, Pars Orbitalis, Pars Triangularis, Rostral Middle Frontal, Superior Frontal, Frontal Pole, Inferior Parietal, Precuneus, Superior Parietal, Supramarginal, Caudal Anterior Cingulate, Posterior Cingulate, Isthmus Cingulate, Rostral Anterior Cingulate, Middle Temporal, Superior Temporal, and Inferior Temporal.

The volume‐weighted mean was calculated according to the following formula:

$$\mathrm{SUV}R_{\mathrm{VW}}=\frac{\sum_{i=1}^{N}\left(SUVR_{i}\;\times \;V_{i}\right)}{\sum_{i=1}^{N}V_{i}}$$

Where $SUVR_{i}$ is the SUVR of region *i*, $V_{i}$ is the corresponding volume of region *i*, and N is the number of regions included.

### 2.5 Assessing cognition

Cognitive performance was assessed using the Repeatable Battery for the Assessment of Neuropsychological Status (RBANS).^59^ This 30‐min battery, which is designed for characterizing cognitive trajectory in older adults, comprises tests of five cognitive domains (with constituent tests of each displayed in parentheses): attention (Digit Span, Coding), immediate memory (List Learning, Story Memory), delayed memory (List Recall, List Recognition, Story Recall, Figure Recall), language (Picture Naming, Semantic Fluency), and visuospatial/constructional ability (Figure Copy, Line Orientation). Further details for each of these subtests can be found in Table S1. These domain scores can be used individually or combined into an omnibus score of general cognitive ability. Raw, non–age‐adjusted scores were used here.

To more accurately characterize individuals experiencing cognitive decline we leveraged the full range of PREVENT‐AD RBANS data collected between 2012 and 2024 (by contrast, neuromelanin MRI data collection began in 2019). We therefore assessed long‐term trajectories of largely retrospective cognitive performance relative to the MRI baseline time point. On average, each participant had 7.54 ± 2.33 RBANS assessments (range: 2–11) over a total of 7.96 ± 1.67 years (range: 5–10). Cognitive data were typically collected within one day of MRI scanning.

### 2.6 Statistical analysis

#### 2.6.1 Assessing within‐ and between‐subject correlates of age and longitudinal change in LC

The linear relationship between LC integrity at baseline and its longitudinal rate of decline was assessed using linear regression using the ‘lm’ function in R v4.4.2, correcting for age, sex, and education.

To test whether longitudinal change in LC was significant within individuals and whether LC integrity at baseline or its change varied by age, we ran a longitudinal mixed effects model defined as:

$$LC_{RI\left(long\right)}∼age_{bl}\times time+sex+edu+1|sub$$

where *age_bl_* denotes age at baseline, *edu* denotes years of education, and *1|sub* represents a random intercept for each subject. *Time* was encoded as the number of months since baseline MRI. LC_RI(long)_ refers to longitudinal LC_RI_, which served as the dependent variable in this model. Main effects on interaction terms are implicitly defined. A significant effect of age at baseline would indicate an overall between‐subject effect of age. A significant effect of time would indicate within‐subject longitudinal change. Finally, a significant age × time interaction would indicate that within‐individual change was different across different ages.

To test whether the age effect was an artifact of incipient AD, we repeated the analysis adding global Aβ burden as an extra interaction term as follows:

$$LC_{\mathrm{RI}\left(\mathrm{long}\right)}∼\;\mathrm{ag}e_{\mathrm{bl}}\times \;\mathrm{time}\;\times \;A\beta_{\mathrm{global}}+\mathrm{sex}+\mathrm{edu}+1|\mathrm{sub}$$

#### 2.6.2 Testing the association between LC integrity and cortical tau deposition

We aimed to test whether LC integrity at baseline (bl‐LC_RI_) or rate of degeneration (slope of longitudinal change in LC_RI_, defined as ΔLC_RI_) was related to neocortical tau deposition, and whether the association was stronger in people with greater Aβ load. ΔLC_RI_ was defined as the β‐coefficient of the regression slope between time and LC_RI_ for each participant. Using SPM12's Multiple Regression framework,^60^ we defined the following model:

$$\begin{array}{ccc} & & \mathrm{Ta}u_{\mathrm{voxelwise}}∼\;\mathrm{bl}{\mathrm{LC}}_{\mathrm{RI}}\times \;A\beta_{\mathrm{global}}+\Delta {\mathrm{LC}}_{\mathrm{RI}}\times \;A\beta_{\mathrm{global}}\\ & & \;+\;\mathrm{ag}e_{\mathrm{bl}}+\;\mathrm{sex}+\mathrm{edu}+\mathrm{Total}\;\mathrm{GMV} \end{array}$$

where *Total GMV* is GM volume, calculated from the CAT12 ‘Segment’ step.^61^ Main effects on interaction terms are implicitly defined. Examining Aβ, bl‐LC_RI_, and ΔLC_RI_ terms in the same model ensures variables control for one another, and main effects can be interpreted in terms of the independent variance explained by each term. In other words, effects of ΔLC_RI_ control for the effects of bl‐LC_RI_ (and other covariates), and vice versa.

The analysis was run across all voxels of the template‐space tau SUVR brain image. Significant clusters of voxels were identified in voxel space using threshold free cluster enhancement ([TFCE], r269) (5000 permutations, *E* = 0.5, *H* = 2.0).^62^ Significant voxels (family‐wise error corrected *p* < 0.05 for robust correction for multiple comparisons) were then projected to surface for presentation in figures. Raw, unthresholded statistical surface maps are shown in Figure S3.

#### 2.6.3 Testing the association between LC integrity and cognition

Finally, we tested whether long‐term trajectories of cognition were predicted by bl‐LC_RI_ or ΔLC_RI_ and whether any such association was modulated by Aβ burden using the following linear mixed‐effects models:

$$\begin{array}{ccc} & & \mathrm{Cognitio}n_{\left(\mathrm{long}\right)}∼\;\mathrm{time}\;\times \;\mathrm{bl}{\mathrm{LC}}_{\mathrm{RI}}\times \;A\beta_{\mathrm{global}}\\ & & \;+\mathrm{time}\;\times \Delta {\mathrm{LC}}_{\mathrm{RI}}\times \;A\beta_{\mathrm{global}}+\;\mathrm{ag}e_{\mathrm{bl}}+\;\mathrm{sex}+\mathrm{edu}+1|\mathrm{sub} \end{array}$$

where the dependent variable is longitudinal cognition for either RBANS total score, or scores of one of the five cognitive subdomains. Main effects on interaction terms are implicitly defined. Cognition_(long)_ refers to cognition being entered into the model as a longitudinal variable, that is, time points were entered individually alongside a separate variable encoding time. Assessing cognition as a longitudinal variable using mixed‐effects models precludes the need to pre‐calculate separate measures for baseline cognition and slope of cognitive change and enables robust assessment of longitudinal trajectories of cognitive change. A main effect of time indicates significant longitudinal change. As with the voxelwise tau model above, all main effects are controlled for other effects in the model, and thus represent independent variance explained.

We report standardized beta (*β*) estimates with 95% confidence intervals (CIs) as well as t‐statistics for all linear‐regression and mixed‐effects models. A threshold of *p* < 0.05 was considered statistically significant. Linear mixed‐effects models were conducted using ‘lmerTest’ v 3.1‐3^63^ in R, with degrees of freedom estimated using the Satterthwaite method (this widely used method calculates degrees of freedom on a per‐effect basis and therefore may vary between fixed effects within a model).

## 3 RESULTS

### 3.1 Longitudinal changes in LC

Across the cohort, bl‐LC_RI_ values averaged 119.7 ± 3.1% and changed by –0.187 ± 0.630% per year. We observed a negative association between bl‐LC_RI_ and longitudinal change in LC (ΔLC_RI_) after correcting for age, sex, and education (*n* = 199, *β* = ‐0.19, CI [–0.32 to –0.05], *t*_194 _= –2.71, *p* = 0.007, Figure 1C). Our mixed‐effects analysis revealed that LC_RI_ declined significantly over time within individuals (*β* = –0.09, CI [–0.13 to –0.06], *t*_336 _= –4.87, *p* < 0.0001), and was lower in older individuals (*β* = –0.14, CI [–0.26 to –0.03], *t*_262 _= 2.01, *p* = 0.045) (Figure 1D). We did not observe a significant age × time interaction (Figure 1E), indicating that rate of LC degeneration did not change as a function of age (*β* = –0.02, CI [–0.06 to 0.02], *t*_342 _= –1.04, *p* = 0.301). No significant differences were observed with sex (*β* = 0.04, CI [–0.08 to 0.15], *t*_256 _= 0.589, *p* = 0.556) or with years of education (*β* = 0.08, CI [–0.04 to 0.20], *t*_257 _= 1.33, *p* = 0.186). The change in LC signal over time was not significantly associated with in‐scanner movement, measured using average frame‐wise displacement during a resting‐state functional MRI run in the same session as the neuromelanin‐sensitive MRI^64^ (Supplementary Analysis). Likewise, LC mask position did not consistently shift between baseline and follow‐up sessions and is therefore an unlikely driver of any changes in signal across the group (Supplementary Analysis).

We next added global Aβ burden as an interaction term to test whether the age effect remained apparent independent of Aβ load. Here we still observed a significant within‐subject longitudinal change (*β* = ‐0.08, CI [–0.12 to –0.04], *t*_307 _= –4.27, *p* < 0.0001), but the between‐subject age effect was no longer apparent (*β* = –0.10, CI [–0.23 to 0.03], *t*_236 _= –1.12, *p* = 0.265). This suggests that part of the apparent age‐related difference in LC_RI_ may be related to variability in Aβ burden. However, there was no significant main effect of global Aβ (*β* = –0.04, CI [–0.16 to 0.07], *t*_386 _= –0.928, *p* = 0.354) on LC_RI_, or any significant interaction between age and Aβ (age × Aβ: *β* = –0.03, CI [–0.13 to 0.08], *t*_369 _= –0.721, *p* = 0.472), indicating that our data do not provide strong evidence that Aβ status moderates age‐related differences in LC degeneration. Further, we found no interaction between age, Aβ, and rate of LC degeneration (age × time × Aβ: *β* = 0.01, CI [–0.03 to 0.05], *t*_325 _= 0.630, *p* = 0.529).

### 3.2 LC integrity at baseline and rate of degeneration independently predict neocortical tau deposition

**FIGURE 2:**
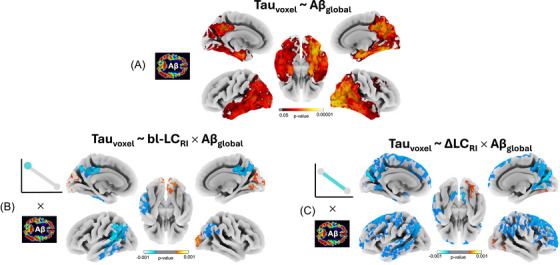
Surface‐projected statistical maps of main effects predicting neocortical tau. (A) Regions where global amyloid (Aβ_global_) predicted local neocortical tau burden. In regions shown in red‐yellow, elevated Aβ_global_ predicted greater neocortical tau. (B) Regions where the bl‐LC_RI_ × Aβ_global_ interaction was statistically significant. In blue regions, lower bl‐LC_RI_ predicted greater tau deposition in people with elevated Aβ_global_. In red‐yellow regions (occipital cortices) greater bl‐LC_RI_ predicted more tau deposition in people with elevated Aβ_global_. (C) Regions where the ΔLC_RI_ × Aβ_global_ interaction is statistically significant. In blue regions, steeper decline in LC_RI_ predicted greater tau deposition in people with elevated Aβ_global_. In red‐yellow regions (occipital cortices) steeper decline in LC_RI_ predicted less tau deposition in people with elevated Aβ_global_. Each main effect is again denoted by a schematic to the left of the respective panel. Throughout the plots, positive associations are denoted in warm colors, and negative associations in cool colors. ”Negative” *p*‐values on color scales indicate *p*‐values of the negative association. All panels show FWE‐corrected TFCE log *p*‐value maps where *p* < 0.05. Unthresholded maps are shown in Figure S3. The main effects of bl‐LC_RI_ and ΔLC_RI_ are not shown as there were no statistically significant voxels.

We observed strong associations between global Aβ burden and voxelwise neocortical tau deposition, with regional specificity in temporal (including parahippocampal gyrus), parietal, and lateral occipital areas (*n* = 178; Figure 2A). Main effects for bl‐LC_RI_ and ΔLC_RI_ did not reveal any regions of significant association with tau burden across the cortex. We did, however, observe significant interactions of these terms with Aβ burden (Figure 2B & C). We interpret this observation as suggesting that in people with greater Aβ burden, both lower bl‐LC_RI_ (Figure 2B) and more negative ΔLC_RI_ (Figure 2C) predict neocortical tau deposition.

Among those with higher global Aβ, low bl‐LC_RI_ predicted greater tau deposition in the lateral temporal and medial parietal cortices, but predicted lower tau deposition in occipital regions. Again in people with higher global Aβ, more negative slopes of ΔLC_RI_ predicted greater tau deposition broadly across lateral temporal cortices (in left hemisphere only), medial and lateral parietal cortices, and across frontal regions. Some sparse areas of the inverse association were also observed in occipital cortices, such that more negative slopes of ΔLC_RI_ predicted lower tau deposition.

These results were robust to various choices in statistical analytic approach and processing including transforming voxelwise tau across individuals to reduce skew, not performing PVC of Aβ or tau, including global Aβ as a binary variable, with positivity defined at a 30‐centiloid (cL) threshold,^65^, ^66^ and including extra covariates of MRI‐PET time delay and hippocampal volume (Figures S4–S8).

### 3.3 LC integrity and degeneration are not directly associated with cortical Aβ deposition

Neither bl‐LC_RI_ nor ΔLC_RI_ predicted voxelwise Aβ SUVR across any region of the cortex (*n* = 178); nor did either predict global Aβ (bl‐LC_RI_: *β* = –0.10, CI [–0.25 to 0.05], *t*_172 _= –1.27, *p* = 0.207; ΔLC_RI_: *β* = 0.03, CI [–0.12 to 0.18], *t*_172 _= 0.370, *p* = 0.712).

### 3.4 LC integrity at baseline and rate of degeneration predict cognitive decline

**FIGURE 3:**
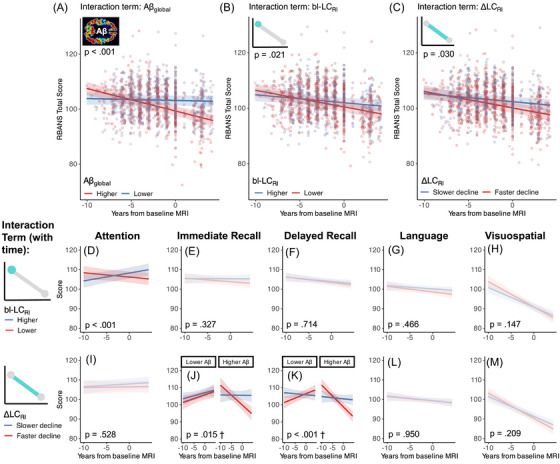
Longitudinal trajectories of cognitive decline modulated by global amyloid and LC integrity. (A–C) Scatterplots showing steeper cognitive decline, measured by RBANS total score in people with (A) elevated global amyloid (Aβ_global_), (B) lower LC integrity at baseline (bl‐LC_RI_), and (C) faster LC degeneration (more negative ΔLC_RI_). Points in A–C show partial residuals from a single linear mixed‐effects model correcting for age at MRI baseline, sex, and education. (D–H) Trendlines showing associations between bl‐LC_RI_ and individual cognitive domain scores. Lower bl‐LC_RI_ was associated with steeper decline of the attention score only. (I–M) Trendlines showing associations between ΔLC_RI_ and individual cognitive domain scores. Lower ΔLC_RI_ was associated with steeper decline of both immediate and delayed memory scores in people with elevated Aβ_global_. Small inset schematics are shown to visually denote the main effects of interest (either Aβ_global_, bl‐LC_RI_ or ΔLC_RI_) in the respective plot groups. The *p*‐values for the interaction between the relevant variable of interest and time are shown on each plot, with J & K showing *p*‐values for the three‐way interaction with amyloid (denoted by †). Note that all variables were modeled continuously, but are plotted here as binary high/low trendlines at 1 SD above or below the mean (± standard error) for visualization purposes only. Colors are numerically reversed in A compared to other plots to align with the notion of “red = worse,” “blue = better.” Colors are faded where the main interaction effect of interest is not statistically significant. Plots are shown faceted by Aβ_global_ if the three‐way interaction between LC × time × amyloid was statistically significant (J & K only). LC = locus coeruleus, RI = relative intensity.

We studied baseline and longitudinal associations between LC integrity and cognitive decline, testing also whether any such associations were stronger in people with elevated global Aβ (*n* = 178; Figure 3). The RBANS total composite score declined significantly over time in the whole cohort (main effect of time: *β* = –0.13, CI [–0.16 to –0.09], *t*_1201 _= –6.92, *p* < 0.0001) but were significantly lower in people with elevated global Aβ at baseline MRI (main effect of Aβ: *β* = –0.10, CI [–0.22 to 0.01], *t*_186 _= –2.94, *p* = 0.004). Cognitive decline was also accelerated in persons with higher global Aβ (time × Aβ interaction: *β* = –0.11, CI [–0.15 to –0.07], *t*_1204 _= –5.35, *p* < 0.0001).

We did not observe any association between overall cognitive performance (RBANS total score) at baseline MRI with bl‐LC_RI_ (*β* = 0.04, CI [–0.08 to 0.16], *t*_184 _= 1.14, *p* = 0.255) or ΔLC_RI_ (*β* = 0.07, CI [–0.04 to 0.19], *t*_190 _= 1.69, *p* = 0.094). We did, however, note steeper trajectories of cognitive decline in people with lower bl‐LC_RI_ (time × bl‐LC_RI_ interaction: *β* = 0.05, CI [0.01 to 0.08], *t*_1217 _= 2.31, *p* = 0.021) and more negative ΔLC_RI_ (time × ΔLC_RI_ interaction: *β* = 0.04, CI [0.00 to 0.08], *t*_1216 _= 2.18, *p* = 0.030). The association between bl‐LC_RI_ and cognitive decline was not stronger for people with elevated global Aβ (time × bl‐LC_RI_ × Aβ interaction: *β* = 0.02, CI [–0.02 to 0.05], *t*_1224 _= 1.03, *p* = 0.304). The association between ΔLC_RI_ and cognitive decline appeared slightly stronger among people with higher global Aβ, but this did not reach statistical significance (time × ΔLC_RI_ × Aβ interaction: *β* = 0.05, CI [0.00 to 0.10], *t*_1236 _= 1.87, *p* = 0.06).

We next tested whether either bl‐LC_RI_ or ΔLC_RI_ predicted longitudinal change in RBANS subscales. Lower bl‐LC_RI_ predicted decline in attention performance (time × bl‐LC_RI_: *β* = 0.06, CI [0.03 to 0.10], *t*_1199 _= 3.544, *p* = 0.0004). More negative ΔLC_RI_ (faster LC degeneration) predicted more negative trajectories of immediate memory (time × ΔLC_RI_: *β* = 0.07, CI [0.02 to 0.12], *t*_1253 _= 2.86, *p* = 0.004), an effect that was stronger in persons with higher global Aβ compared to those with lower global Aβ (time × ΔLC_RI_ × Aβ: β = 0.08, CI [0.02 to 0.15], *t*_1280 _= 2.42, *p* = 0.016). More negative ΔLC_RI_ slopes also predicted faster declines in delayed memory in people with elevated global Aβ (time × ΔLC_RI_ × Aβ: β = 0.14, CI [0.08 to 0.20], *t*_1272 _= 4.307, *p* < 0.0001). Language and visuospatial scores were not related to either bl‐LC_RI_ or ΔLC_RI_. Full statistics for these models are shown in Tables S2 and S3.

## 4 DISCUSSION

In a cohort of older adults with a familial history of AD, we examined longitudinal changes in LC integrity measured in vivo using neuromelanin‐sensitive MRI. We observed lower LC integrity in older individuals and provide evidence that incipient AD pathology may contribute to this effect. Among participants with higher global Aβ, both lower LC integrity and a faster rate of LC degeneration were independently associated with greater neocortical tau deposition in regions corresponding to Braak stages IV/V. At the same time, lower LC integrity and faster degeneration were associated with less tau deposition in occipital regions, suggesting a specificity of tau deposition pattern to LC degeneration. Finally, we showed independent associations between LC integrity and rate of LC degeneration with long‐term cognitive trajectories, such that LC baseline integrity was associated with changes in attention, whereas rate of LC degeneration was associated with memory decline. Our results support the *neuromodulatory fragility hypothesis* ^7^ and the role of LC in AD pathogenesis.

### 4.1 LC age‐related degeneration may be partly influenced by incipient AD pathology

We observed a robust within‐individual decline in LC integrity over a ≈4‐year follow‐up period. This was accompanied by a group‐level negative association with age, whereby the older individuals (≈80 years of age) had lower LC integrity than younger participants (≈60 years of age). We did not observe an increasing rate of LC degeneration with advancing age, in keeping with one previous study,^46^ but in contrast to another.^49^ The discrepancy may reflect our explicit control for baseline LC integrity in longitudinal models.

The association between LC integrity and LC degeneration was weak (*β* ∼–0.2 according to our data) but should nonetheless be noted. This negative association was unexpected and may reflect a floor effect, whereby those with lower integrity have less to lose, or longer‐term life trajectories of neuromelanin accumulation, where lower LC_RI_ may indicate a “younger” LC that has not yet reached its lifetime peak neuromelanin level, and may therefore increase over time rather than decline.^39^, ^44^ Regardless, the effect size was small and warrants replication.

Age‐related LC decline has been reported, with neuronal count decreasing by 20%–40% across the lifespan (see review^21^). Neuromelanin‐sensitive MRI studies report an inverted‐U–shaped lifespan trajectory, with LC signal peaking at around age 60.^39^, ^40^, ^41^, ^42^ However, these exclusively cross‐sectional studies do not explicitly test whether there is a robust decline in older age or whether the trajectory is better described as a plateau. Indeed many studies focusing on older adults report no age effect.^13^, ^16^, ^17^, ^67^ Where age effects are reported, it is often unclear whether LC changes reflect “healthy aging” or incipient AD, as these studies rarely exclude individuals with AD pathology elsewhere in the brain (see^68^ for one exception). In our analyses, adjusting for global Aβ removed the group‐wise association with age, whereas within‐individual decline remained significant. This pattern is consistent with the possibility that age‐related LC differences partly reflect incipient AD pathology; however, we did not observe strong direct associations between LC integrity and Aβ, either globally or voxelwise, so this connection requires further study.

### 4.2 LC integrity at baseline and rate of LC degeneration independently predict neocortical tau

We found that both lower LC integrity and faster rate of LC degeneration were independently associated with greater neocortical tau deposition in people with higher global Aβ burden. In persons with elevated Aβ, associations between cross‐sectional LC integrity and neocortical tau were localized to medial parietal and left lateral temporal cortices, whereas associations between LC rate of degeneration and neocortical tau were more widespread, including frontal cortices. These regions correspond roughly to Braak stages IV/V.^2,3^ Notably, we did not observe a significant association localized to entorhinal or parahippocampal cortices after accounting for global Aβ. The pattern is similar to a previous study in which LC integrity predicted tau deposition primarily in posterior cortices in people with autosomal‐dominant AD.^47^ Our observed pattern is more extensive than some reports of exclusively medial‐temporal localized associations between LC neuromelanin signal and tau SUVR.^45^, ^46^ Another study measured tau directly in LC, showing associations with neocortical tau in people with higher global Aβ, with effects localized to Braak stage III/IV regions.^69^ Thus, although there is some variability across findings, the presence of an association between LC measures and neocortical tau in individuals with likely incipient AD appears robust.

Our data are consistent with the increasingly supported model that the LC facilitates the spread of tau to the neocortex, thereby driving AD progression. In this model, the LC seeds tau initially to other neuromodulatory nuclei including the cholinergic basal forebrain, followed by the medial temporal lobes.^5^, ^6^, ^7^, ^70^, ^71^, ^72^, ^73^ Later, when interacting with Aβ‐induced neuronal hyperexcitability, tau spreads more rapidly and broadly across the neocortex.^74^, ^75^, ^76^, ^77^, ^78^, ^79^ According to our data and prior work,^45^, ^47^, ^69^ existing neuroimaging tools reliably capture LC degeneration in vivo, and this degeneration occurs prior to clinically detectible cognitive decline but after substantial neocortical tau spread. Moreover, within‐individual rates of LC degeneration seem particularly indicative of widespread tau pathology in later‐stage *Braak* regions. Our findings therefore support longitudinal in vivo measurement of LC integrity as a marker of tau spread and pathological extent. Direct measurement of tau with PET provides greater specificity but is invasive, substantially more expensive and less repeatable.

This model may primarily describe the canonical pattern of tau deposition in AD. However, increasing evidence indicates that AD is unlikely to follow a single trajectory, but instead comprises distinct subtypes.^80^, ^81^, ^82^, ^83^ These subtypes vary in tau distribution (e.g., temporal‐ vs posterior‐predominant) and clinical presentation (e.g., memory‐ vs visual‐predominant). We report an unexpected association whereby greater LC degeneration was related to less tau in occipital cortices. Our findings lead us to hypothesize that a posterior‐predominant tau subtype may have a partially non–LC‐related etiology. This reversed pattern was not observed in a previous analysis of LC integrity as a predictor of neocortical tau deposition in people with autosomal‐dominant AD,^47^ a condition more likely to follow a unified pathological trajectory than sporadic AD. Further investigation and explicit testing of this hypothesis are warranted.

### 4.3 LC integrity at baseline and rate of LC degeneration independently predict changes in cognition

We found that individuals with lower LC integrity at baseline and those with a faster rate of LC degeneration exhibited steeper cognitive decline over a ≈14‐year period, assessed mostly retrospectively. This effect was independent of global Aβ burden. As with our findings on neocortical tau distribution, this demonstrates distinct and additive associations of both LC integrity and rate of LC degeneration with cognitive decline. These data are consistent with previous studies showing associations linking LC health to cognition^45^, ^49^, ^67^ and support the notion that LC neuronal density may confer cognitive reserve and is therefore critical for maintaining cognitive function in older age (for a review see Ref. 21).

We observed that baseline LC integrity predicted the rate of attentional change over time, in line with the established role of LC in sustaining and modulating attention.^21^, ^22^, ^23^, ^24^, ^25^ This finding is consistent with previous work linking lower LC integrity to reduced practice effects^84^ and with a model whereby LC integrity confers cognitive reserve.^30^, ^31^ Variation in LC integrity appears to correspond with the ability to maintain effective attention in cognitively unimpaired older adults.^21^, ^23^, ^24^, ^30^, ^31^ We also found that the rate of LC degeneration predicted memory decline (both immediate and delayed recall), and that this effect was strongest in people with higher Aβ burden, in keeping with our hypothesis. This supports the notion that those exhibiting significant LC degeneration are most likely to be in preclinical stages of AD, which are characterized early by selective memory impairment.^32^, ^85^ One previous study reported a similar effect whereby LC integrity moderated the association between memory change and Aβ but did not examine specific tests of attention.^45^ To our knowledge, ours is the first study to report associations of LC integrity and its rate of change with separate cognitive subdomains. We note that we lacked the statistical power to fully compare the strength of LC associations across subdomains directly. Nonetheless, our findings support LC integrity and degeneration as markers that may help to differentiate individuals in preclinical stages of AD from those on a healthy aging trajectory.

## 5 CONCLUSIONS

The LC may be the earliest system directly involved in AD pathogenesis. With tools that can probe LC health and neocortical tau and Aβ in vivo, we are now able to characterize and track earlier stages of AD than what was possible using purely histological methods. We hope that our study encourages additional research using longitudinal in vivo measurement of this important neuromodulatory system. Future work should assess whether improving LC health can produce downstream effects on disease outcomes.^86^ Furthermore, specificity of LC degeneration should be explored in relation to atypical AD subtypes and other neurodegenerative diseases. Finally, a better understanding of the cholinergic, serotonergic, orexinergic, and dopaminergic systems, including how they interact, spread tau, and support compensatory mechanisms, is essential to support and expand the *neuromodulatory fragility hypothesis*,^7^ with the ultimate goal of developing a complete understanding of AD pathogenesis. This line of research may ultimately support the development of a new generation of highly effective disease‐modifying treatments.

## CONFLICT OF INTEREST STATEMENT

Authors have no competing interests to declare. Author disclosures are available in the Supporting Information.

## CONSENT STATEMENT

All human subjects in this study provided informed written consent prior to participation.

## Supporting information




**Supporting Information**: alz71427‐sup‐0001‐tableS1‐S3.docx

**Supporting Information**: alz71427‐sup‐0002‐figureS1‐S8.docx

**Supporting Information**: alz71427‐sup‐0003‐SuppMat.docx

**Supporting Information**: alz71427‐sup‐0004‐SuppMat.pdf
