# Supplementary material for: Locus coeruleus degeneration is associated with cortical tau deposition and cognitive decline in older adults at familial risk of Alzheimer's disease
Source: Alzheimers Dement. 2026 Apr 24;22(4):e71427. doi: 10.1002/alz.71427 (PMC13108248; doi:10.1002/alz.71427)
Supplement: Supplementary file 1 — Supporting Information: alz71427‐sup‐0001‐tableS1‐S3.docx [file ALZ-22-e71427-s002.docx]

Table S1 | Descriptions of the subtests for the Repeatable Battery for the Assessment of Psychological Status (RBANS).

| Subtest name | Cognitive Domain | Brief description |
| --- | --- | --- |
| List Learning | Immediate Memory | Ten semantically unrelated words are read aloud; the participant recalls as many as possible immediately across four trials; total correct responses are summed. |
| Story Memory | Immediate Memory | The participant hears a short story and immediately recalls it; scoring is based on correctly recalled story units. |
| Figure Copy | Visuospatial/ Constructional | The participant copies a complex geometric figure; scoring reflects accuracy and spatial placement of elements. |
| Line Orientation | Visuospatial/ Constructional | The participant matches the orientation of two lines to a reference array; score is the number of correct matches. |
| Picture Naming | Language | The participant names pictured everyday objects; score is the number of correctly named items. |
| Semantic Fluency | Language | The participant names as many items as possible from a given category within 60 seconds; score is total correct responses. |
| Digit Span | Attention | The participant repeats sequences of digits forward and backward; score reflects the number of correctly reproduced sequences. |
| Coding | Attention | The participant matches numbers to symbols using a key, completing as many correct pairings as possible within 60 seconds. |
| List Recall | Delayed Memory | After a delay, the participant freely recalls words from the List Learning task; score is the number of correct words. |
| List Recognition | Delayed Memory | The participant identifies List Learning words from a list containing targets and distractors; score reflects correct recognitions and discrimination accuracy. |
| Story Recall | Delayed Memory | After a delay, the participant recalls the story; scoring is based on correctly recalled story units. |
| Figure Recall | Delayed Memory | After a delay, the participant draws the previously copied figure from memory; scoring reflects accuracy and placement of elements. |

Table S2 | Model statistics for the relationship between LC degeneration and cognitive decline. Each outcome variable was run as a separate model, with all main effects of interest listed. Bolded and italicised main effect titles indicate primary hypotheses. Statistics shown in bold highlight significant (p<0.05) effects.

| Main effects | Outcome variable | t-value | p-value | df |  | Outcome variable | t-value | p-value | df |
| --- | --- | --- | --- | --- | --- | --- | --- | --- | --- |
| Time | RBANS Total | **-6.92** | **0.000** | **1201** |  | Delayed Recall | **-3.60** | **0.000** | **1224** |
| bl-LC |  | 1.14 | 0.255 | 184 |  |  | 0.42 | 0.675 | 188 |
| LC-slope |  | 1.69 | 0.094 | 190 |  |  | 1.77 | 0.078 | 199 |
| Amyloid |  | **-2.94** | **0.004** | **186** |  |  | **-4.45** | **0.000** | **191** |
| ***bl-LC*Time*** |  | **2.31** | **0.021** | **1217** |  |  | 0.37 | 0.714 | 1247 |
| ***LC-slope*Time*** |  | **2.18** | **0.030** | **1216** |  |  | 1.68 | 0.093 | 1246 |
| Amyloid*Time |  | **-5.35** | **0.000** | **1204** |  |  | **-5.86** | **0.000** | **1229** |
| bl-LC*Amyloid |  | 0.40 | 0.691 | 190 |  |  | 0.08 | 0.938 | 199 |
| LC-slope*Amyloid |  | 0.84 | 0.403 | 200 |  |  | 1.83 | 0.068 | 215 |
| ***bl-LC*Amyloid*Time*** |  | 1.03 | 0.304 | 1224 |  |  | 0.31 | 0.760 | 1256 |
| ***LC-slope*Amyloid*Time*** |  | 1.87 | 0.062 | 1236 |  |  | **4.31** | **0.000** | **1272** |
| Time | Attention | 1.09 | 0.275 | 1186 |  | Language | **-2.75** | **0.006** | **1273** |
| bl-LC |  | 0.99 | 0.323 | 179 |  |  | 1.55 | 0.123 | 216 |
| LC-slope |  | 0.72 | 0.472 | 184 |  |  | 0.15 | 0.882 | 237 |
| Amyloid |  | -1.35 | 0.177 | 180 |  |  | -0.87 | 0.385 | 224 |
| ***bl-LC*Time*** |  | **3.54** | **0.000** | **1199** |  |  | 0.73 | 0.466 | 1296 |
| ***LC-slope*Time*** |  | 0.63 | 0.528 | 1198 |  |  | -0.06 | 0.950 | 1294 |
| Amyloid*Time |  | **-3.83** | **0.000** | **1189** |  |  | -0.84 | 0.400 | 1278 |
| bl-LC*Amyloid |  | -0.71 | 0.477 | 184 |  |  | 0.88 | 0.377 | 238 |
| LC-slope*Amyloid |  | 0.21 | 0.836 | 190 |  |  | 0.70 | 0.485 | 268 |
| ***bl-LC*Amyloid*Time*** |  | 1.00 | 0.320 | 1204 |  |  | 1.70 | 0.090 | 1304 |
| ***LC-slope*Amyloid*Time*** |  | -1.47 | 0.142 | 1213 |  |  | 0.65 | 0.518 | 1316 |
| Time | Immediate Recall | -1.12 | 0.265 | 1239 |  | Visuospatial/Constructional | **-11.76** | **0.000** | **1246** |
| bl-LC |  | 1.16 | 0.246 | 190 |  |  | -0.09 | 0.925 | 199 |
| LC-slope |  | **2.57** | **0.011** | **201** |  |  | 0.69 | 0.489 | 214 |
| Amyloid |  | **-2.83** | **0.005** | **193** |  |  | -1.33 | 0.185 | 204 |
| ***bl-LC*Time*** |  | 0.98 | 0.327 | 1255 |  |  | 1.45 | 0.147 | 1271 |
| ***LC-slope*Time*** |  | **2.86** | **0.004** | **1253** |  |  | 1.26 | 0.209 | 1268 |
| Amyloid*Time |  | **-5.23** | **0.000** | **1236** |  |  | -0.65 | 0.518 | 1251 |
| bl-LC*Amyloid |  | 1.12 | 0.266 | 201 |  |  | 1.04 | 0.300 | 214 |
| LC-slope*Amyloid |  | 1.31 | 0.192 | 219 |  |  | -0.19 | 0.850 | 236 |
| ***bl-LC*Amyloid*Time*** |  | 1.70 | 0.090 | 1264 |  |  | -0.83 | 0.408 | 1280 |
| ***LC-slope*Amyloid*Time*** |  | **2.43** | **0.015** | **1280** |  |  | -0.06 | 0.949 | 1295 |

Table S3 | Interpretation guide for main effects listed in Table S2.

| Main Effect | Interpretation/Meaning |
| --- | --- |
| Time | Does this cognitive score change over time? |
| bl-LC | Do cognitive scores at time 0 differ according to bl-LC? |
| LC-slope | Do cognitive scores at time 0 differ according to LC-slope? |
| Amyloid | Do cognitive scores at time 0 differ according to global amyloid? |
| bl-LC*Time | Does rate of cognitive change depend of bl-LC? |
| LC-slope*Time | Does rate of cognitive change depend on LC-slope? |
| Amyloid*Time | Does rate of cognitive change depend on global amyloid? |
| bl-LC*Amyloid | Is any dependence of cognition at time 0 on LC-bl stronger in people with more global amyloid? |
| LC-slope*Amyloid | Is any dependence of cognition at time 0 on LC-slope stronger in people with more global amyloid? |
| bl-LC*Amyloid*Time | Is any dependence of the rate of cognitive change on LC-bl stronger in people with more global amyloid? |
| LC-slope*Amyloid*Time | Is any dependence of the rate of cognitive change on LC-slope stronger in people with more global amyloid? |
