# Supplementary material for: Locus coeruleus degeneration is associated with cortical tau deposition and cognitive decline in older adults at familial risk of Alzheimer's disease
Source: Alzheimers Dement. 2026 Apr 24;22(4):e71427. doi: 10.1002/alz.71427 (PMC13108248; doi:10.1002/alz.71427)
Supplement: Supplementary file 2 — Supporting Information: alz71427‐sup‐0002‐figureS1‐S8.docx [file ALZ-22-e71427-s004.docx]

**Methods: ROIs templates in standard space**


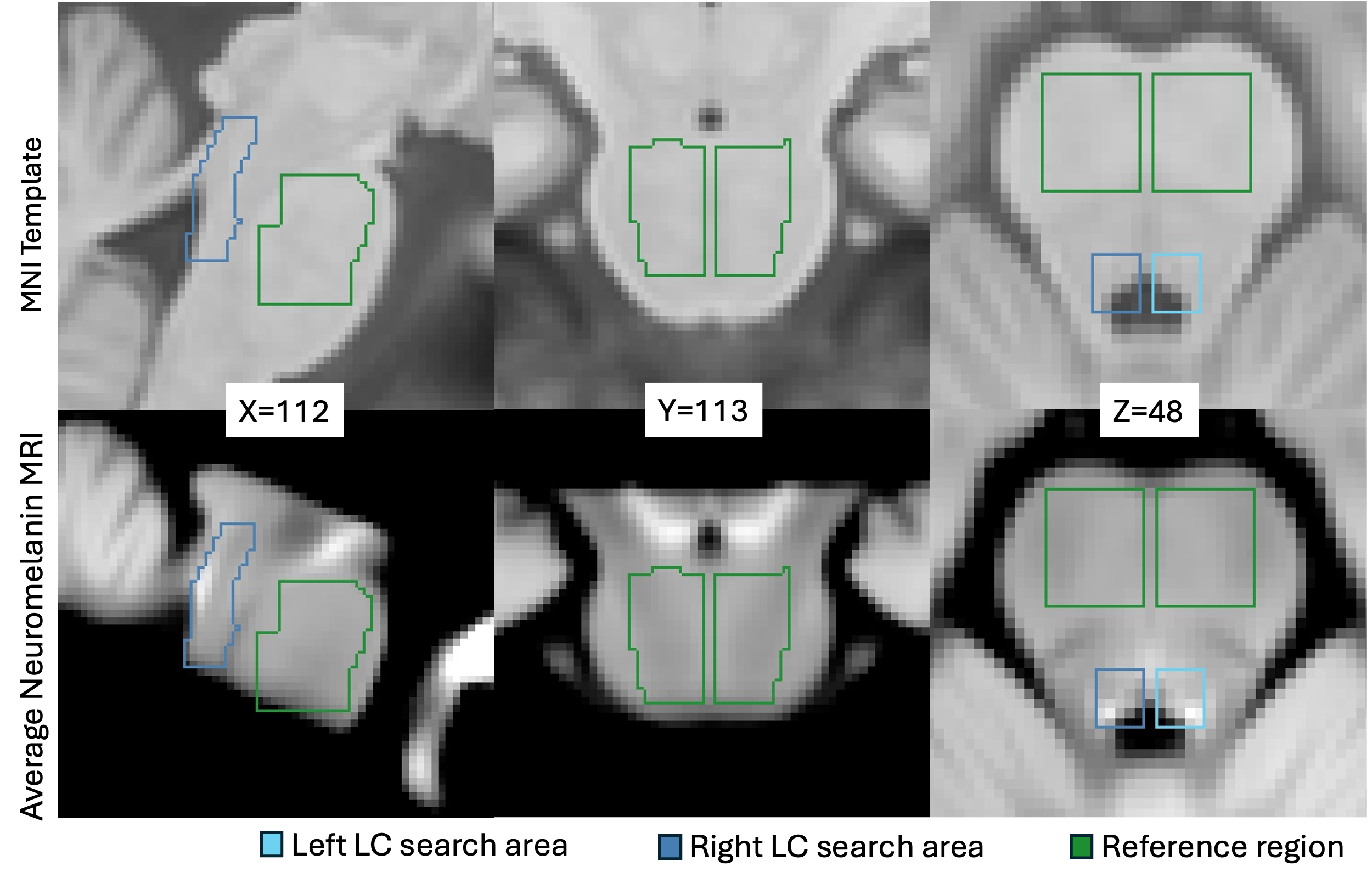


Fig. S 1 | Standard space masks of locus coeruleus (LC) search areas and reference region for relative intensity calculation. Masks are aligned with (and, here, overlaid on) the MNI 2009a T1w standard space brain (upper panel) or the neuromelanin MRI averaged across subjects in MNI space (lower panel). The LC search areas were designed to encompass an area surrounding the entire rostro-caudal length of the LC. More precise individualised localisation of LC voxels was based on a neuromelanin MRI signal intensity within this search area. LC voxels were defined as being the 10 brightest contiguous voxels within the search area. On the neuromelanin MRI, the LC hyperintensity is clearly visible falling within the LC search space. Likewise, the Substantia Nigra hyperintensity is clearly visible rostral to (and outside of) the reference region search space).

**Testing baseline vs. follow-up LC mask placement**


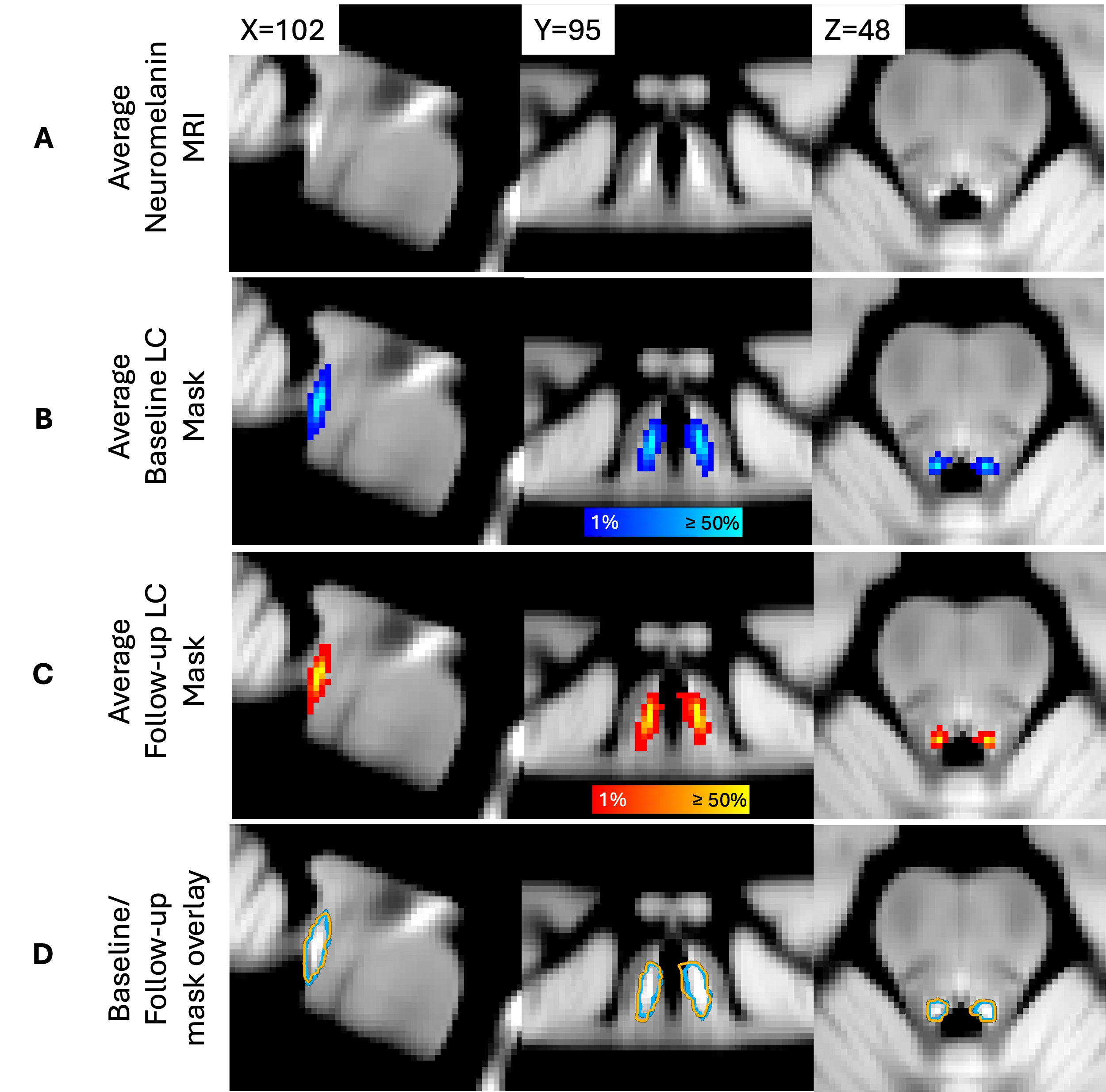


Fig. S 2 | Average locus coeruleus mask placement at baseline and follow-up. Panel A shows the subject-averaged standard space neuromelanin MRI, with LC hyperintensity visible along the edge of the fourth ventricle. Panel B and C show the same image, with the average LC mask at baseline sessions (B) or the latest follow-up session (C) overlaid. The colour of the mask represents the proportion of participants who have a mask voxel in that space. Panel D shows an outline of each of the masks shown in B and C (with linear interpolation) overlayed on the same image. This image shows no apparent consistent shift in mask placement over time that might have contributed in any meaningful way to the findings of the study. All images are aligned in MNI 2009a standard-space in sagittal, coronal and axial views (left to right) with MNI coordinates shown at the top of the image.

Fig. S 3 | Raw, unthresholded p-values of the effects shown in the main body of manuscript (Fig. 2). Top row: Main effect of global amyloid; Middle row: Main effect of bl-LC * global amyloid interaction term; Bottom row: Main effect of ΔLC * global amyloid interaction term.

Fig. S 4 | Raw, unthresholded p-values and significant TFCE, FWE-corrected clusters of the same analysis as presented in Fig. 2 of the main paper, with the exception that voxelwise tau value were first log-transformed in order to reduce the effect of outlying values on the observed effect. Top row: Main effect of global amyloid; Middle row: Main effect of bl-LC * global amyloid interaction term; Bottom row: Main effect of ΔLC * global amyloid interaction term.

Fig. S 5 | Raw, unthresholded p-values and significant TFCE, FWE-corrected clusters of the same analysis as presented in Fig. 2 of the main paper, with the exception that amyloid and tau SUVR values were not corrected for partial volume correction (PVC). This step is often recommended but sometimes omitted as can introduce bias. For completion, we explored not conducted PVC, but see a largely similar cluster pattern. Top row: Main effect of global amyloid; Middle row: Main effect of bl-LC * global amyloid interaction term; Bottom row: Main effect of ΔLC * global amyloid interaction term.

Fig. S 6 | Raw, unthresholded p-values and significant TFCE, FWE-corrected clusters of the same analysis as presented in Fig. 2 of the main paper, with the exception that partial volume correction was not applied and amyloid variables were entered as a binary measure, thresholded at 30 centiloids. This threshold is recommended as an upper limit for certainty of abnormal amyloid levels (Collij et al. 2024, Alzheimers Dement). Top row: Main effect of global amyloid; Middle row: Main effect of bl-LC * global amyloid interaction term; Bottom row: Main effect of ΔLC * global amyloid interaction term


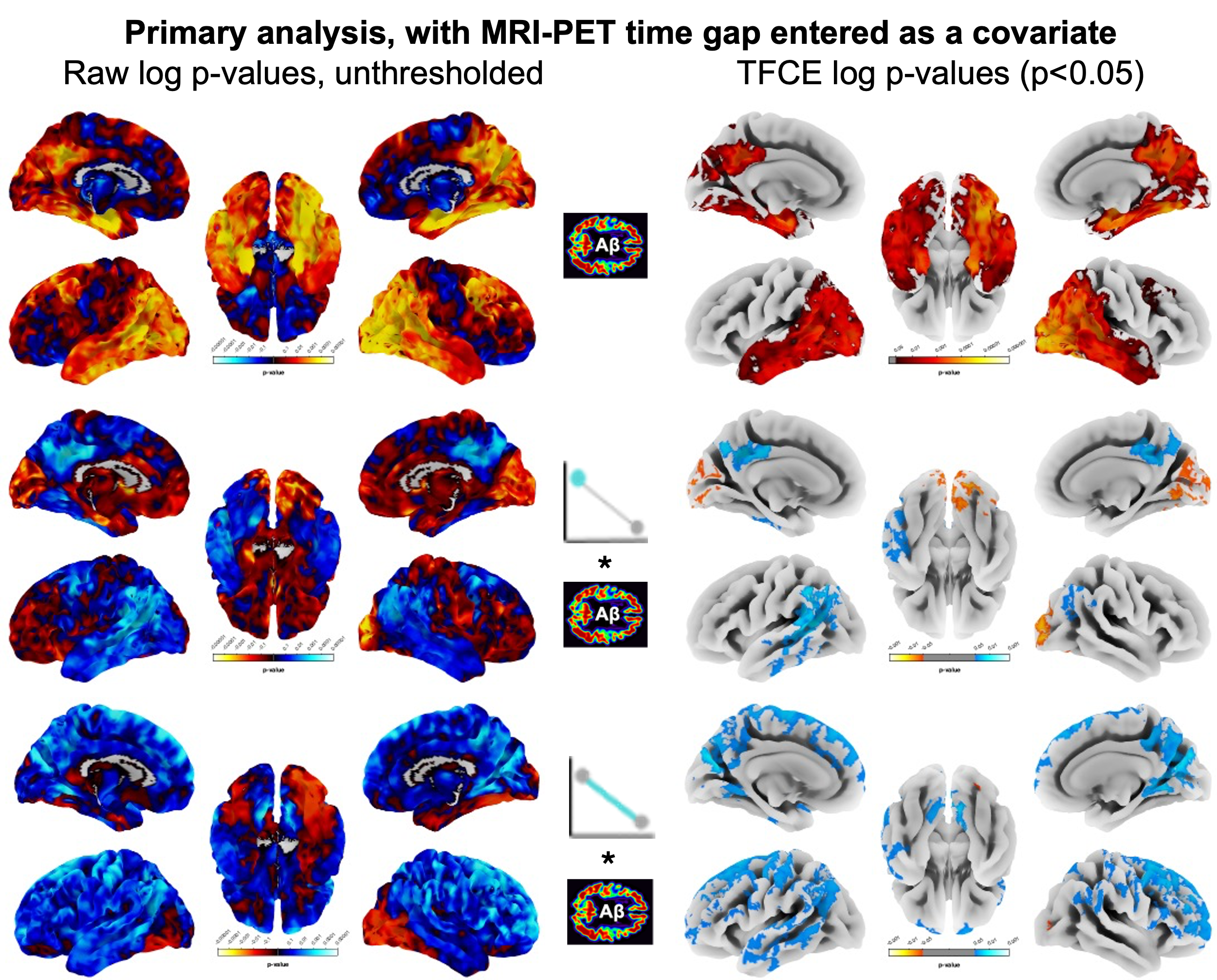


Fig. S 7 | Raw, unthresholded p-values and significant TFCE, FWE-corrected clusters of the same analysis as presented in Fig. 2 of the main paper, with the exception that the time delay between baseline MRI and PET data collection was entered as a covariate. Little to no difference in the main effects are visible speaking to the robustness of our effects to this variation. Top row: Main effect of global amyloid; Middle row: Main effect of bl-LC * global amyloid interaction term; Bottom row: Main effect of ΔLC * global amyloid interaction term


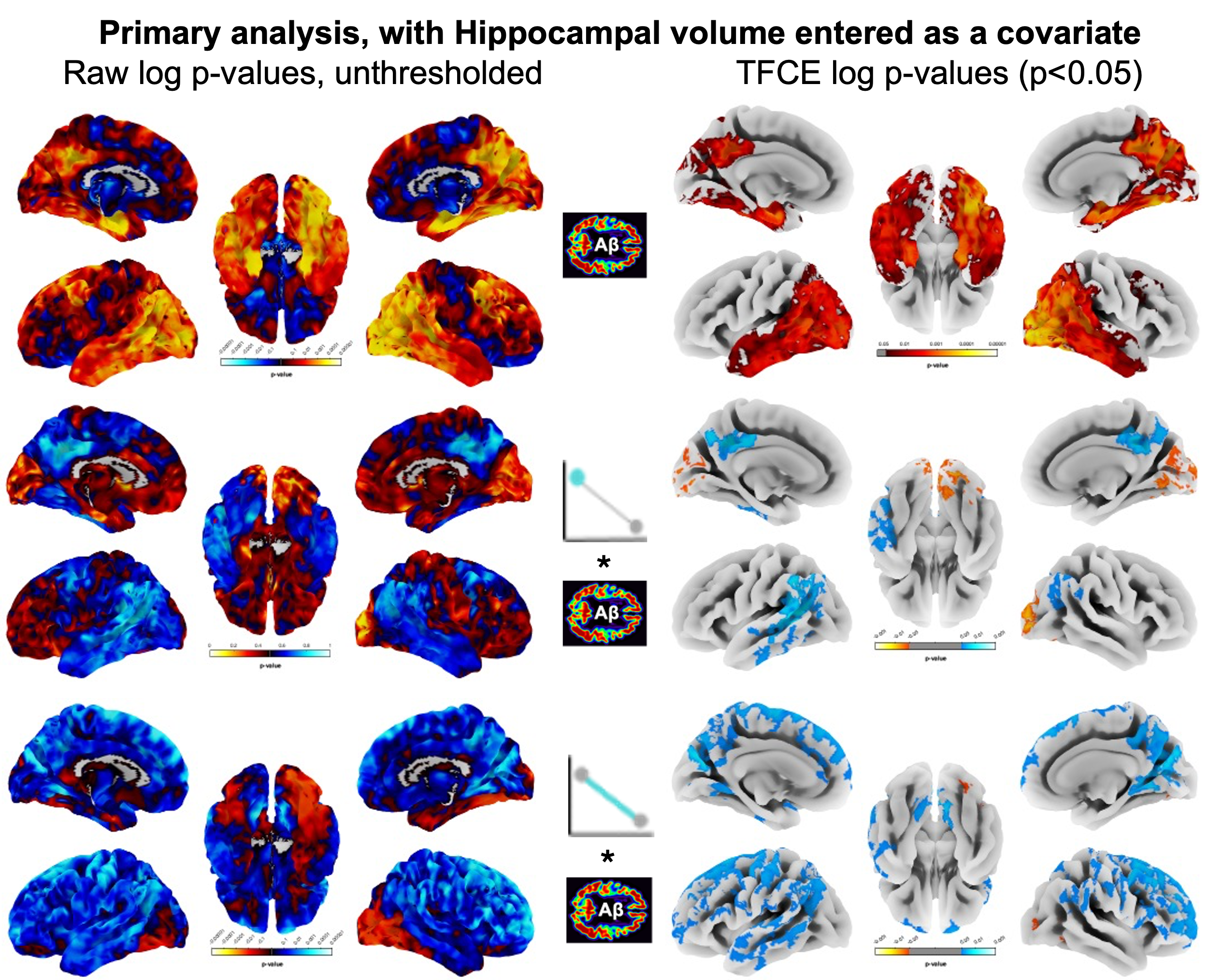


Fig. S 8 | Raw, unthresholded p-values and significant TFCE, FWE-corrected clusters of the same analysis as presented in Fig. 2 of the main paper, with the exception that hippocampal volume (from freesurfer v7.4.2 was entered as a covariate. Little to no difference in the main effects are visible speaking to the robustness of our effects to this variation. Top row: Main effect of global amyloid; Middle row: Main effect of bl-LC * global amyloid interaction term; Bottom row: Main effect of ΔLC * global amyloid interaction term
