# Supplementary material for: Locus coeruleus degeneration is associated with cortical tau deposition and cognitive decline in older adults at familial risk of Alzheimer's disease
Source: Alzheimers Dement. 2026 Apr 24;22(4):e71427. doi: 10.1002/alz.71427 (PMC13108248; doi:10.1002/alz.71427)
Supplement: Supplementary file 3 — Supporting Information: alz71427‐sup‐0003‐SuppMat.docx [file ALZ-22-e71427-s001.docx]

**Movement is not significantly associated with change in LC_RI_**

It is feasible that increased in-scanner movement over time could decrease signal-to-noise and artifactually cause apparent lower measures of LC_RI_. In order to exclude movement as a source of variance in LC_RI_ change we calculated framewise displacement of a resting-state functional MRI (fMRI) collected in all participants in the same session as the neuromelanin-sensitive MRI.

*Scan parameters for multi-echo resting-state fMRI*:  3mm^3^, TR=1000 ms, TEs=12/30.11/48.22 ms, FA=50°, TA=10:24.

Using *fsl_motion_outliers* we calculated the average framewise displacement across the entire run (echo #2) for every session in order to get a proxy measure for movement, under the assumption that movement is a trait that would be comparable across the entire MRI session. This measure of framewise displacement was entered as a covariate in a linear mixed effects model that assessed the change in LC_RI_ over time in our cohort:

$$\mathrm{LC}_{RI(long)} \sim time framewise displacement+\mathrm{ag}_{\mathrm{bl}}+sex+edu+1|sub$$

The main effect of time remained statistically significant (β=-0.09, CI[-0.12, -0.05], t_335_=-4.43, p<0.0001), and there was no significant association between framewise displacement and LC_RI_ (β=-0.05, CI[-0.12, 0.01], t_485_=-1.49, p=0.137) nor any significant interaction with time (β=0.01, CI[-0.03, 0.05], t_341_=0.39, p=0.695). These results strongly suggest that changes in LC_RI_ over time in our study were not significantly influenced by in-scanner movement.”

**The LC mask placement does not vary consistently across time**

An apparent change in LC signal over time could be attributed to a consistent shift in LC mask placement between baseline and follow-up scans. For example, if follow-up scans consistently mask more rostral parts of the LC than baseline scans across participants, then signal changes may be masked or amplified.

In order to test whether any shift was present across any spatial dimension, we calculated the centroid in mm of each MNI-space bilateral mask either at baseline or subject’s latest follow-up scan using *fslstats <LC mask> -C*. We then subtracted the baseline position from follow-up position for all subjects for each spatial dimension, and ran one-sample t-tests comparing the groupwise shift to zero.

We observed no significant effects in any dimension, suggesting that any variation in mask placement across time was due to random variation (x: t_198_=0.069, p=0.945; y: t_198_=-1.157, p=0.249; z: t_198_=0.113, p=0.910.
